# Early palliative radiation versus observation for high-risk asymptomatic or minimally symptomatic bone metastases: study protocol for a randomized controlled trial

**DOI:** 10.1186/s12885-020-07591-w

**Published:** 2020-11-17

**Authors:** Daniel B. Rosen, Cory D. Benjamin, Joanna C. Yang, Connor Doyle, Zhigang Zhang, Chris A. Barker, Max Vaynrub, T. Jonathan Yang, Erin F. Gillespie

**Affiliations:** 1 Department of Radiation Oncology, Memorial Sloan Kettering Cancer Center, 1275 York Ave, Box 22, New York, NY 10065 USA; 2 Department of Epidemiology and Biostatistics, Memorial Sloan Kettering Cancer Center, New York, NY USA; 3 Department of Surgery, Orthopaedic Service, Memorial Sloan Kettering Cancer Center, New York, NY USA; 4 Center for Health Policy and Outcomes, Memorial Sloan Kettering Cancer Center, New York, NY USA

**Keywords:** Bone metastasis, Radiation therapy, Skeletal-related events (SRE), Patient-centered outcomes

## Abstract

**Background:**

In patients with metastatic cancer, the bone is the third-most common site of involvement. Radiation to painful bone metastases results in high rates of pain control and is an integral part of bone metastases management. Up to one-third of inpatient consults are requested for painful bone metastases, and up to 60% of these patients had evidence of these lesions visible on prior imaging. Meanwhile recent advances have reduced potential side effects of radiation. Therefore, there is an opportunity to further improve outcomes for patients using prophylactic palliative radiation to manage asymptomatic bone metastases.

**Methods/study design:**

In this trial, 74 patients with metastatic solid tumors and high-risk asymptomatic or minimally symptomatic bone metastases will be enrolled and randomized to early palliative radiation or standard of care. This will be the first trial to assess the efficacy of prophylactic palliative radiation in preventing skeletal related events (SREs), the primary endpoint. This endpoint was selected to encompass patient-centered outcomes that impact quality of life including pathologic fracture, spinal cord compression, and intervention with surgery or radiation. Secondary endpoints include hospitalizations, Bone Pain Index, pain-free survival, pain-related quality of life, and side effects of radiation therapy.

**Discussion:**

In this study, we propose a novel definition of high-risk bone metastases most likely to benefit from preventive radiation and use validated questionnaires to assess pain and impact on quality of life and health resource utilization. Observations from early patient enrollment have demonstrated robustness of the primary endpoint and need for minor modifications to Bone Pain Index and data collection for opioid use and hospitalizations. With increasing indications for radiation in the oligometastatic setting, this trial aims to improve patient-centered outcomes in the polymetastatic setting.

**Trial registration:**

ISRCTN Number/Clinical trials.gov, ID:NCT03523351. Registered on 14 May 2018.

**Supplementary Information:**

The online version contains supplementary material available at 10.1186/s12885-020-07591-w.

## Background

### Radiation therapy for treatment of bone metastases

Recent advances in radiation therapy (RT) have resulted in improved tumor control and fewer side effects, contributing to extending the duration and improving the quality of life of patients with metastatic disease [1, 2]. Despite these advances, metastatic disease, and in particular metastatic disease in bone, represents a major source of cancer-related death [3]. Additionally, metastatic bone lesions can decrease a patient’s quality of life and overall functioning due to local sequelae including: spinal cord compression, pathological fractures, and acute or chronic pain. RT is widely utilized as an effective palliative treatment for painful bone metastases, but currently is only applied once lesions become symptomatic [4–6].

### Rationale for study timing of early radiation

Studies have shown that early palliative care improves overall survival and quality of life for patients with metastatic cancer [7, 8]. While multiple studies have evaluated RT for treatment of symptomatic bone lesions [9–12], the impact of early, upfront RT for asymptomatic or minimally symptomatic (non-opioid dependent) bone metastases has not been evaluated, and has the potential to improve patient-centered outcomes.

To understand patterns of inpatient radiation oncology care, a single institution inpatient radiation oncology consult registry was created and analyzed [13]. Of 1151 inpatient consults between July 2015 and June 2016, 319 consults (28%) were for evaluation of symptomatic bone metastases, and two-thirds went on to receive RT for pain management. The median survival of all patients seen in consultation was 4 months; 9% percent of patients discontinued RT to transition to hospice care, 8% died before the end of planned RT. Importantly, 61% of patients treated for painful bone metastases had imaging confirming presence of the lesion within 4 months prior to RT. While RT is an effective treatment option for palliation, it appears that RT is often being delivered too late in the disease course. The current trial seeks to understand if it is beneficial for patients with asymptomatic or minimally symptomatic metastatic disease to receive early, upfront radiation treatment.

### Skeletal-related events

The clinical consequences of bone metastases include pain and pathologic fracture, which can greatly impact the general quality of life for patients with metastatic cancer. Skeletal related events (SREs) are defined as pathological fractures, spinal cord compression, palliative radiotherapy for bone pain, orthopedic surgery for bone pain, or orthopedic surgery for fracture prevention or treatment. As a result, SREs serve as a rational composite endpoint to guide development of therapies to prevent clinically significant consequences from bone metastases. Data for rates of SREs in patients with metastatic disease primarily come from trials of medical therapy (i.e. bisphosphonates) and range from 50 to 70% at 1–2 years, which is reduced to 40–50% in patients taking these bone-modifying agents [14, 15]. Another study found that the median time to SRE in patients who had SREs was 155 days, with an overall rate of 48% at 21 months [16]. On prospective trials, patients undergoing conventional RT for symptomatic bone metastases have fracture rates around 5% [9]. Retrospective studies have shown that RT significantly reduces the risk of SREs in patients with asymptomatic bone metastases [17]. Until the current study, there have been no prospective trials to address the possible benefits of RT to asymptomatic or minimally symptomatic high-risk bone metastases in reducing the risk of the development of SREs.

### Defining “high-risk” bone metastases

In a retrospective study completed at our institution [13], we found that the most commonly treated sites of bone metastases in inpatients with clinically significant pain were in the spine (51%), joints such as hip and shoulder (11%) and long bones such as femur and humerus (11%). Additionally, we incorporated lesions that met partial criteria for pathological fracture indicating surgical intervention according to the Mirels classification [18] (location and 1/3–2/3 cortical thickness) and Spinal instability neoplastic score (SINS) [19] (junctional spine and posterior involvement). Thus, we propose to define the high-risk bone lesions as detailed below in *Eligibility*.

### Risk of hospitalization

In a recent multicenter, observational study designed to describe cross-regional differences in health resource utilization of SREs in Europe and US, Durah et al. found that 25% of reported SREs required inpatient hospitalization for a mean of 18 days [20]. Furthermore, 96% of the SREs resulted in inpatient and/or outpatient procedures. In another study, 26% of SREs were associated with inpatient hospital stay with mean duration of 19.5 days [21]. Interventions that prevent the development of SREs have been shown to significantly reduce costs associated with SREs [22, 23], due to the subsequent reduction in hospital stay and more complicated post-SRE procedures. Therefore, SREs result in considerable health resource utilization, and impose a substantial financial burden. This study will investigate the impact of upfront radiation therapy on the number of hospitalizations related to SREs in patients with high-risk bone metastases.

### Study rationale and innovation

The current standard of care of radiation for symptomatic bone lesions was established by multiple randomized trials in the 1980s. In the intervening 4 decades, the introduction of modern systemic therapies has improved the prognosis of patients with metastatic cancer. Additionally, side effects from radiation therapy have been reduced by increasingly targeted techniques. Hence, the practice of withholding RT until metastatic lesions prove sufficiently painful may be outdated. There are several theoretical benefits to early, upfront RT to asymptomatic or minimally symptomatic bone metastases, such as reducing the risk for SREs. Furthermore, early palliative RT may reduce the risk of developing painful bone metastases and improve pain-free survival and quality of life. Finally, there are significant direct and indirect costs associated with hospitalizations for painful bone metastases. This trial proposes the evaluation of a new treatment paradigm, in which bone metastases are treated with upfront RT in the outpatient setting, before they become symptomatic. Given ongoing trials addressing all sites of metastatic disease with radiation in the oligometastatic setting, we opted to focus on the polymetastatic setting (defined as greater than 5 sites of metastatic disease).

## Methods/study design

### Study setting

This is a single-institution randomized (1:1) phase II trial enrolling patients with metastatic solid tumors at Memorial Sloan Kettering Cancer Center. We plan to recruit 74 patients in total to the protocol.

### Study duration

It is anticipated that patients will be accrued to the study over 24 months. Once enrolled, patients will continue on the protocol until 12 months have elapsed, or until an SRE occurs. Recruitment began in May 2018 and is ongoing.

### Inclusion criteria

Eligibility criteria of the patient/subject population

1. Histologically confirmed solid tumor malignancy with polymetastastic spread (greater than 5 sites of metastatic disease) detected on cross-sectional imaging
2. Has high-risk bone metastasis (es) that is (are) asymptomatic or minimally symptomatic (not requiring opioids). High-risk bone metastases are defined as meeting any of the following criteria:
  - Bulky site of disease in bone (≥ 2 cm)
  - Disease involving the hip (acetabulum, femoral head, femoral neck), shoulder (acromion, glenoid, humeral head), or sacroiliac joints
  - Disease in long bones with1/3–2/3 cortical thickness (humerus, radius, ulna, clavicle, femur, tibia, fibula, metacarpals, phalanges),
  - Disease in vertebrae of the junctional spine (C7-T1, T12-L1, L5-S1) and/or disease with posterior element involvement. Bone metastases that are within 3 cm of each other will be treated as one site.
3. ECOG performance status 0–2
4. Age ≥ 18 years
5. Ability to provide informed consent
6. Patients of reproductive age must agree to practice an effective contraceptive method.

### Exclusion criteria

Criteria that will exclude patients from the study:

1. Previous RT to the target treatment site(s) that precludes the development of a treatment plan that respects normal tissue tolerances
2. Serious medical co-morbidities that preclude RT
3. Women who are pregnant or lactating
4. Target lesion(s) is/are complicated bone metastases that include clinical or radiological evidence of spinal cord compression or impending long-bone pathological fracture (by Spinal instability neoplastic score and Mirels criteria, respectively)
5. Leptomeningeal disease
6. Malignant pleural effusion
7. Absolute neutrophil count (ANC) < 1.0 K/mcL and platelet count < 50 K/mcL at the time of enrollment
8. If entry to the trial will cause clinical delays in their treatment management (e.g. if systemic or surgical therapy is warranted and trial entry would delay this)

### Intervention

The intervention in this study is early, upfront radiation therapy to asymptomatic or minimally symptomatic (defined as high-risk bone metastases). Up to 5 boney lesions per patient may be enrolled.

#### Radiation therapy (RT) technique

For patients assigned to receive RT to the lesion(s) enrolled on this study, options for radiation dose and fractionation schedule will follow institutional standards for symptomatic bone metastases (See Table 1). Accounting for the patient’s global clinical status, the treating radiation oncologist should select a radiation regimen that will result in optimal lesion local control while not exceeding local normal tissue radiation tolerance.

**Table 1:** Standard options for radiation dose, fractionation, and verification imaging

Total Dose	Fractions	Dose per Fraction	Verification Imaging
800cGY	1	800cGY	MV or KV
2000cGY	5	400cGY	MV or KV
3000cGY	10	300cGY	MV or KV
3000cGY	5	600cGY	KV and CBCT
3500cGY	5	700cGY	KV and CBCT
2400cGY	3	800cGY	KV and CBCT
2700cGY	3	900cGY	KV and CBCT
2400cGY	1	2400cGY	KV and CBCT

Of note, any technique may be used, including conventional, 3D-CRT, or IMRT, with appropriate image verification per the treating radiation oncologist.

Patients will be simulated using appropriate immobilization as determined by the treating radiation oncologist. This will be followed by acquisition of a CT scan in the treatment position. The use of intravenous or oral contrast will be at the discretion of the treating radiation oncologist.

The treating radiation oncologist must delineate the gross tumor volume (GTV). Additional clinical target volume (CTV) and planning target volume (PTV) should be included for highly conformal treatments including IMRT and SBRT and are defined in Table 2.

**Table 2:** Target volume definitions

Target volume	Definition	MSKCC Standard Approach
GTV	Gross tumor in the bone, including soft tissue extension	Consider fusing diagnostic imaging including PET. MRI is recommended for spinal metastases. Consider reviewing diagnostic and/or simulation imaging with a diagnostic radiologist.
CTV	Margin for microscopic extension of tumor	GTV + 5 mm but not extending beyond the bone unless direct extension with adjacent tissue invasion is present. NOTE: For lesions in the spine undergoing SBRT, published guidelines will be followed [24]
PTV	Margin for setup error (patient positioning and treatment delivery)	CTV + 3 mm (if using daily CBCT)

Organs at risk (OAR) of radiation toxicity should be contoured according to institutional standards. In general, this includes OARs adjacent to the target lesion as well as those at risk of radiation exposure, and therefore may commonly include several organs (such as bowel) in patients undergoing intensity-modulated radiation therapy (IMRT) or stereotactic body radiation therapy (SBRT).

Dose constraints for radiation treatment planning will be followed according to institutional standards and will depend on the radiation technique and total dose prescribed.

### Trial design

**Fig. 1:**
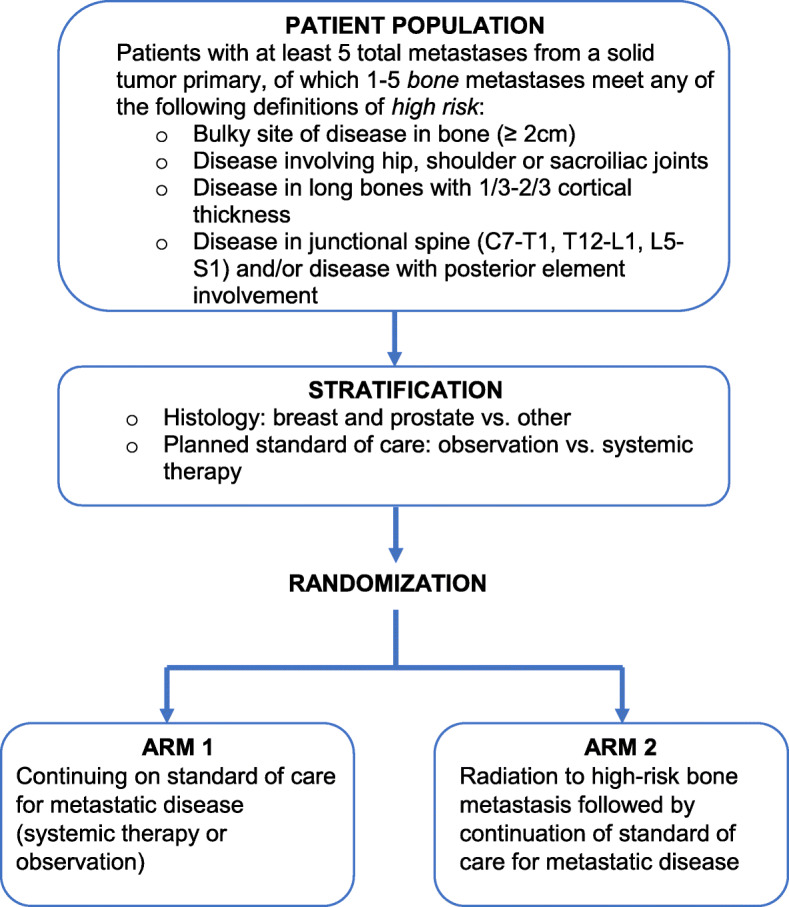
Study Schema

Subjects will be randomized to receive either standard of care (**Arm 1**) or early radiation therapy (**Arm 2**) followed by standard of care (see Fig. 1). The standard of care arm will include observation or systemic therapy, per treating medical oncologist discretion.

Each patient will be stratified by their disease histology (breast and prostate vs. other) and planned standard of care (observation vs. systemic therapy), as determined by the treating medical oncologist. Randomization will be conducted using random permuted block. Clinical research associates will assign patients to interventions based on the random permuted block.

Randomization will be completed on a *per patient* basis, even though each patient may have up to 5 eligible sites of metastatic disease. At the time of randomization, physicians will fill out the Lesions Identification Worksheet (Additional file 1) to ensure clarity in the number and location of metastatic lesions under study.

Any protocol amendments must be approved by the relevant IRB and subsequently disseminated to investigators, consenting physicians and research personnel.

#### Arm 1

Patients enrolled to Arm 1 will receive appropriate therapy (systemic therapy or observation) as determined by their medical oncologist. Systemic therapy may include continuation of the current systemic therapy or initiation of a new systemic therapy. Per the protocol, patients in Arm 1 will be able to receive palliative RT to progressed, painful lesions (an SRE) only at the time of symptom development or determination of high risk for fracture requiring surgical intervention. Upfront preventive RT that is not considered part of standard of care is not allowed.

#### Arm 2

Patients enrolled on Arm 2 of the study will receive upfront palliative RT selectively to 5 or fewer high-risk bone metastases (as defined in Inclusion and listed in Fig. 1).

### Trial objectives and endpoints

#### Primary objective and endpoint

To assess whether early palliative radiation of the high-risk asymptomatic or minimally symptomatic bone metastases in patients with metastatic cancer can decrease the number of SREs, defined as pathological fractures, spinal cord compression, or palliative radiotherapy and orthopedic surgery to bone.

#### Secondary objectives and endpoints

- To compare the number of hospitalizations related to SREs between standard of care and upfront RT arms.
- To compare pain-related quality of life of between standard of care and RT arms, using
  - the Brief Pain Inventory (BPI) form.
- To collect health care utilities in the standard of care and RT arms using the EuroQol
  - Group EQ-5D-5L form.
- To compare pain-free survival (PFS) and overall survival (OS) between RT and standard of care arms.
- To evaluate adverse events in the upfront RT arm using CTCAE v4.0 toxicity.

**Treatment Evaluation** (See Table 3 for summary and timeline of assessments).

**Table 3:** Study schedule for enrollment and assessment

Timeframe	Pre-registration	Within 4 weeks of study entry	Once every 5 treatment days during radiation	N months following randomization +/− 4 weeks			Within 1 week of SRE^c^
				3 mo	6 mo	12 mo	
Standard of care biopsy of a metastatic lesion or pathology review confirming metastatic cancer	X						
CBC with differential		X		X	X	X	X
CMP		X					
Imaging studies^d^		X		X^a^	X^a^	X^a^	X^a^
History and physical		X		X^b^	X^b^	X^b^	X^b^
Performance status		X		X	X	X	X
Adverse Events evaluation (CTCAE v4.0)			X	X	X	X	X
BPI short form		X	X	X	X	X	X
EQ-5D-5L		X		X	X	X	X
Lesions Identification worksheet		X					

^a^Follow-up imaging at discretion of treating physician. The same imagining modality is encouraged for assessment between time points

^b^Patient’s height not needed at follow-ups and SRE

^c^All assessments following an SRE are optional, but highly recommended

^d^Baseline imaging can be obtained within 6 weeks of study entry

#### Pre-treatment

- Standard of care biopsy of any metastatic lesion or pathology review documenting confirmed metastatic disease.

Within 4 weeks (28 days) of study entry:

- CBC with differential
- Comprehensive metabolic panel (CMP=Na, K, Cl, CO2, BUN, Creatine, Ca, Glucose, total protein, albumin, alkaline phosphatase, total bilirubin, AST and ALT)
- Complete medical history including current medications, comorbidities and performance status.
- Physical exam including weight, height and vital signs (O2 saturation, blood pressure, heart rate, respiratory rate, and temperature)
- BPI short form and EQ-5D-5L
- Lesion Identification Worksheet completed by the enrolling physician(s)

During Treatment/Intervention:

All patients in Arm 2 (**RT arm**) will be assessed for toxicities according to CTCAE v 4.0 and pain score using BPI short form once every 5 treatment days. Only patients in Arm 2 (**RT arm**) will have radiation treatment related adverse event evaluation. Following randomization, all patients in Arm 1 and Arm 2 will be scheduled for follow-up at 3 months (+/− 4 weeks), 6 months (+/− 4 weeks), 12 months (+/− 4 weeks), and receive the following assessments:

- CBC with differential
- Imaging studies (Follow-up imaging at the discretion of the treating physicians)
- History and physical
- Performance status
- Adverse event evaluation (CTCAE V 4.0) (Excluding Arm 1 patients)
- Brief Pain Inventory (BPI) Short Form
- EQ-5D-5L

As noted in Table 3, if an SRE occurs, the assessments should be completed within 1 week, which may not be feasible. If the patient is unable to come in for a follow-up appointment within 1 week, a telephone follow-up will suffice, and the CBC with differential, history and physical, performance status and imaging studies may be deferred. If the patient has had any of these assessments completed locally, records should be obtained to fulfill these requirements. If the attending physician is able, the adverse event evaluation can be completed over the phone.

Additionally, the BPI Short Form and EQ-5D-5L can be completed over the phone or sent to the patient via mail, fax or electronic mail by the physician, physician office assistant or research staff. For SRE, it is preferred that the BPI Short Form and EQ-5D-5L are completed over the phone if patients are unable to come in for a follow-up appointment. If the patient cannot be reached by phone or prefers to complete the questionnaires personally, the questionnaires will be sent via mail, fax or electronic mail. The questionnaires sent directly to patients must be blank forms with no patient identifiers, only study ID numbers. If questionnaires are sent via mail, the patient must be provided with a pre-filled business envelope that will allow patients to return it with no expense. Patients must also have the option of returning questionnaires by electronic mail or fax. If a patient is unable to come in for a follow-up appointment or complete the questionnaires over the phone, the method by which questionnaires are sent to patients and the method by which questionnaires are returned will be determined based on the patient’s preference or if they are unable to be contacted by phone, the contact information on file for the patient as some patients may not have access to a phone, fax or electronic mail.

### Primary endpoint assessment

An SRE in this protocol is defined as any pathologic fractures, spinal cord compression, and/or palliative radiotherapy and orthopedic surgery to bone.

- Pathologic fracture: Defined clinically or radiographically.
- Spinal cord compression: A classification scheme was developed at our institution to further categorize an objective definition of spinal cord compression [25]. For the purposes of this study, ESCC2 meets the definition of a spinal cord compression.
- Intervention with surgery or radiation: The decision for intervention should be based on clinical judgment per standard of care and is at the discretion of the treating physicians.

SREs will be assessed by the SRE Assessment Form (Additional file 2).

### Secondary endpoint assessment

#### Hospitalizations

Patients on both arms will be followed for number and duration of hospitalizations for SREs as a surrogate for health care cost.

#### Brief pain inventory short form (BPI)

All patients enrolled will have their cancer-specific pain assessed using the Brief Pain Inventory (BPI) Short Form questionnaire at baseline, 3 months, 6 months, 12 months and optimally within 1 week of an SRE. (See Additional file 3 for the questionnaire). The BPI is a validated patient questionnaire where patients self-report the severity of their pain and how it affects their functioning [4]. The questionnaire asks patients to rate their highest, lowest, average, and current pain intensity on 0 to 10-point scale (0 being no pain and 10 being the most pain). Of note, we added a cover letter to the questionnaire so patients would only report pain from the lesion(s) enrolled in the study (See Additional file 4 for cover letter). In addition to pain scores, patients are asked to report their perceived effectiveness of their current treatment, and rate the degree that pain interferes with general activity, mood, walking ability, normal work, relations with other persons, sleep, and enjoyment of life also on a 10-point scale (0 being no interference and 10 being the most interference). The BPI also asks what pain medications the patient uses for their pain and how much relief they feel that they are receiving from the medication (0% being no relief and 100% being complete relief).

#### Pain-free survival

Patients enrolled on both arms of the study will also be included in an analysis of pain-free survival, defined as time from study entry to start of opioids or death. To accurately capture patients’ reasons for opioid use, we added the following question to the SRE assessment form (Additional file 2): “Is the participant taking any opioid medication for pain relating to lesion(s) enrolled?” Of note, patients on opioid use at study enrollment (for a non-index lesion) will not be included in this analysis.

#### EuroQol EQ-5D-5L

All patients enrolled will have their overall health utility assessed using the EuroQol EQ-5D-5L questionnaire at baseline, 3 months, 6 months, 12 months and optimally within 1 week of an SRE. (See Additional file 5 for the questionnaire). Patients who are randomized to Arm 2 will complete the form prior to receiving RT. The EuroQol EQ-5D-5L questionnaire is a standardized instrument created by the EuroQol Group to measure a patient’s health utility [26, 27] and is used to reflect global quality of life. In brief, EuroQol EQ-5D-5L assesses 5 dimensions (5D) of health: mobility, self-care, usual activities, pain/discomfort, and anxiety/depression. Patients are asked to rate each dimension on 5 levels (5 L): 1-no problems, 2-slight problems, 3-moderate problems, 4-severe problems, and 5-extreme problems. The results in each dimension are combined into a 5-digit code, which is then transformed to an index value from 0 to 1 (sometimes called preference values, utilities, or QALY weights) based on country-specific value sets provided by the EuroQol Group. For the current study, the United States value set will be used to obtain the overall index values and are available at http://www.euroqol.org/about-eq-5d/valuation-of-eq-5d/eq-5d-5l-value-sets.html. The index value has interpretive anchors at 0 (dead) and 1 (best possible health). In order to minimize missing values on the overall index value, missing values are given a numeric value of 9; ambiguous values (i.e. two responses to the same question) are also given a numeric value of 9 and are accounted for in the published overall index values.

Patients enrolled on both arms of the study will also be included in an analysis of overall survival, defined as time from study entry to death.

### Adverse event reporting

The National Cancer Institute (NCI) Common Terminology Criteria for Adverse Events (CTCAE) version 4.0 will be used to evaluate and report toxicities. Adverse events will depend on the sites receiving RT and the normal tissues adjacent to those sites. Patients undergoing RT commonly experience fatigue within the first several months of treatment. Other RT toxicities will depend on the region treated as well as the regimen. Of note, pathologic fracture can occur as a side effect of radiation [28], further emphasizing the importance of comparing radiation to no radiation in the upfront setting. If surgery is needed on previously radiated bone, patients can also be at higher risk for infection.

After informed consent is signed, study site personnel will record the occurrence and nature of each patient’s pre-existing conditions, including clinically significant signs and symptoms of the disease. During the study, site personnel will record any change in the pre-existing condition(s), and the occurrence and nature of any new adverse events, according to the timeline in Table 3.

All AEs related to protocol procedures are reported. All AEs occurring after the patient receives the first dose of radiation therapy must be reported in regard to their assessment of the potential relatedness of each AE to protocol procedure, studied disease state, and/or radiation modality via a case report form (CRF). If a patient’s radiation treatment is discontinued as a result of an AE, personnel must clearly report the circumstances and data leading to any dosage reduction or discontinuation of treatment.

Events leading to the clinical outcome of death due to disease progression will be included as part of the safety and efficacy analyses for this study. If a death is considered related to treatment, the death should be reported as a Serious Adverse Event (SAE) and appropriate guidelines followed for SAE reporting. Any clinically significant findings from labs, vital sign measurements, and other procedures should be reported as well.

See Additional file 6 for details of Adverse Event definitions, process of attribution to radiation therapy, and follow-up.

### Safety assessment

With the help of the research study assistant (RSA), the principal investigator will review each case at the time of enrollment to verify eligibility. The RSA will work with the principal investigator to ensure that the protocol is followed carefully.

The Data and Safety Monitoring (DSM) Plans at Memorial Sloan Kettering Cancer Center were approved by the National Cancer Institute in September 2001. The plans address the new policies set forth by the NCI in the document entitled “Policy of the National Cancer Institute for Data and Safety Monitoring of Clinical Trials” which can be found at: http://cancertrials.nci.nih.gov/researchers/dsm/index.html. The DSM Plans at MSKCC were established and are monitored by the Office of Clinical Research.

There are several different mechanisms by which clinical trials are monitored for data, safety and quality. There are institutional processes in place for quality assurance (e.g., protocol monitoring, compliance, and data verification audits, therapeutic response, and staff education on clinical research QA) and department procedures for quality control. In addition, there are two institutional committees that are responsible for monitoring the activities of our clinical trials programs, including the Data and Safety Monitoring Committee (DSMC) for phase I and II clinical trials, which reports to the Center’s Research Council and Institutional Review Board.

Deviations (prospective/retrospective) are submitted in the Protocol Information Management.

System (PIMS) and reviewed by the Institutional Review Board (IRB).

### Statistical considerations

The primary objective of this study is to compare the rate of skeletal related events.

(SRE) from the date of randomization to death or 12 months, whichever occurs first, between patients who received standard of care versus upfront, early palliative RT to high-risk bone metastases. We expect 60–80% of the enrolled patients can be followed for 1 year. An SRE is defined as pathological fractures, spinal cord compression, or palliative radiotherapy and orthopedic surgery for bone pain. Data suggest that the event rate is around 60% in the standard of care arm [14, 15, 18]. In our institutional experience, 75% of inpatient radiation consultation led to palliative radiation for painful bone metastasis, an SRE. Furthermore, 61% of these lesions were diagnosed at least 4 months prior to undergoing RT.

#### Sample size

The primary endpoint is a binary variable and the rate refers to proportion and is defined as the number of lesions that had SRE divided by the total number of target lesions. This analysis is lesion-based so an SRE (or no SRE) at one site does not affect the status of other sites from the same patient. We estimate that the investigational arm has the event rate around 30%. Radiation therapy is extremely effective in alleviating pain due to bone metastases with 70–80% pain control. By preventing the development of significant bone pain, which often leads to SREs, RT can effectively reduce SREs. To this end we will analyze at least 66 patients with valid SRE endpoint (randomized 1:1 to each arm) to achieve > 80% power in detecting such a difference using a two-sample, one-sided proportion test with alpha< 0.05. Since patients who withdraw before the endpoint can be evaluated will not be included in the analysis (i.e., it will NOT be an intent-to-treat analysis) we will over-accrue to account for withdrawals to ensure a minimum of 33 patients in each arm (subject to stratification, see *Section 15.2*) who can contribute the analyzable endpoint of SRE. We believe an additional 10% would be sufficient so totally we expect to randomize 74 patients. A small portion of patients (< 15%) may have multiple lesions, in which case they will be treated as independent analysis units. In other words, the eventual effect sample size may be slightly higher than 66 because this objective will be analyzed per lesion. Of note, palliative radiation therapy is a well-established, frequently used treatment for patients with metastatic disease. It is often the standard of care for patients with symptomatic metastatic lesion. It is extremely unlikely to be more toxic or cause earlier deaths.

We expect to enroll all 74 patients within 2 years.

Secondary objectives will be analyzed per patient. Unless otherwise specified, all endpoints are defined within the time window from the date of randomization to death or 12 months, whichever occurs first. To compare the number of hospitalizations related to SREs between standard of care and upfront, early palliative RT arms we will employ a Wilcoxon rank sum test.

To compare quality of life between standard of care and RT arms, using the Brief Pain Inventory (BPI) and EuroQol Group EQ-5D-5L forms, we will test the difference (between the two arms) of the survey results at the following time points: 3 months, 6 months, 12 months and optionally but recommended within 1 week of any SRE. The individual quantitative scores derived from the BPI, and the utility scores as well as the overall health scores derived from EQ-5D-5L, will be summarized at these assessment times using descriptive statistics (means and standard deviations, medians and quartiles). Differences between the two arms in terms of these quantitative scores at various time points of interest will be evaluated for both statistical and clinical significance using Wilcoxon rank sum tests and established minimally important differences (MIDs) for the various measures, respectively. For scale scores with no established MIDs, the “half standard deviation” rule will be applied (i.e., differences of a half standard deviation will be considered clinically significant). At the conclusion of the study data at each time point will be presented, the number of patients in each group at a given time point will be documented, and the mean EQ-5D-5L and BPI scores for each group will be plotted over time and longitudinal pattern will be examined with the possibility of proposing more complicated regression methods such as the linear mixed models. The categorical answers (e.g., YES vs NO) from EQ-5D-5L will be compared between the two arms using Fisher’s test at each time point as well and odds ratios together with confidence intervals will be computed. Other non-quantifiable answers (e.g., treatment receiving for pain) will be summarized descriptively. For the last question in BPI, the score for pain interference on the BPI Short Form is the mean of the 7 interference questions as long as at least 4 are completed.

For comparing pain-free survival and overall survival between the two arms, both of which are time to event endpoint (time from randomization to start of opioids or death which is not necessarily within 12 months from randomization), we will use log-rank test.

To evaluate CTCAE v4 toxicity events in the upfront RT arms, we will tabulate all toxicities and summarize the CTCAE v4.03 scores and present descriptive statistics. This will be done at 3 months, 6 months, and 12 months from randomization.

## Discussion

Clinical trials of radiation therapy in the setting of bone metastases have historically emphasized palliation, defined as improvement in pain, function, and quality of life after treatment of symptomatic lesions. Alternatively, medical therapy with bone-modifying drugs (such as bisphosphonates) have generally assessed efficacy of preventing complications of bone metastases. In evaluating our institutional inpatient experience, we discovered that for a majority of patients with bone metastases eventually requiring palliative RT, the lesions were identifiable on systemic imaging performed prior to developing symptoms (within 4 months) [13]. This potential window of intervention prompted us to design a prospective randomized controlled trial to evaluate the potential for prophylactic RT to metastatic bone lesions from solid tumors to improve patient outcomes—thus, for the first time evaluating palliative RT as prophylactic treatment for bone metastases.

The selection of the primary endpoint serves as a unique element of this trial. In the setting of asymptomatic or minimally symptomatic bone lesions, the most common prior endpoint for radiation trials in treatment of bone metastases – pain control – would not suffice. Other common endpoints for RT trials include local control and disease-free survival, however these have more limited utility in the metastatic setting. Furthermore, RT to bone metastases is not without toxicity, including bone fractures particularly in the spine after stereotactic body radiotherapy (SBRT) [19]. With the goal to develop a robust patient-centered outcome, we therefore selected skeletal related events (SREs), which incorporates pathologic fractures (either from disease or treatment), cord compression, and radiation or surgical intervention. Furthermore, SREs are supported by retrospective data in the setting of radiation for asymptomatic bone metastases [17] and commonly used in the prophylactic setting for prospective trials of medical therapy [29].

Patient selection is another important factor in developing a trial that can facilitate future implementation into routine practice, if findings are positive. We therefore developed and will be testing a novel definition of high-risk bone metastases (as defined above in *Eligibility*) that is supported by retrospective evidence from our institution [13].

To better understand the potential clinical significance of prophylactic palliative radiation for asymptomatic bone metastases, validated patient-centered and health services-related secondary endpoints include pain (measured with a modified BPI), quality of life (measured with EuroQOL EQ-5D-5L), and hospitalizations (a surrogate for health resource utilization). With global BPI observed to be less useful than a lesion-specific BPI, the tool was modified using a cover letter (Additional file 4). This lesion-specific questionnaire will likely be useful for future trials of radiation in setting of bone metastases. And lastly, with the expanding nature of our MSKCC Regional Network across 3 states, we integrated follow-up questions about recent hospitalizations to record location, presenting complaint, and admission duration.

This trial has several important limitations. First, inclusion of all solid tumor histologies will improve likelihood of rapid accrual, but prevent analyses of tumor response differentiated according to primary tumor type. Second, the inclusion of radiation as an SRE is limited due to physician discretion perhaps including use of RT in scenarios where radiation is not standard of care, such as ESCC 1 in spine (epidural extension but not spinal cord compression). Nonetheless, enrolling physicians will review each potential radiation-related SRE with the PI and details of rationale for each SRE will be tracked.

Ultimately, we are optimistic that this study may reveal an additional therapeutic window for patients with metastatic disease to bone. By studying patients with polymetastatic disease, this trial complements ongoing work among patients with oligometastatic disease, where locally consolidative RT has demonstrated progression-free and overall survival benefits [30, 31].

### Trial status

At the time of writing (January 2020), 40 patients have been accrued (out of 74 planned).

## Supplementary Information

**Additional file 1.** Lesion Identification Worksheet.

**Additional file 2.** SRE Assessment Form.

**Additional file 3.** Brief Pain Inventory (BPI) Short Form questionnaire (hyperlink and licensing information).

**Additional file 4.** Cover letter for patient QoL packet.

**Additional file 5.** EuroQol EQ-5D-5L questionnaire (hyperlink and licensing information).

**Additional file 6.** Adverse Event definitions, process of attribution to radiation therapy, and follow-up.

## Data Availability

Data sharing is not applicable to this article as no datasets were generated or analyzed during the current study. Institutional professionals, investigators, and departments on the IRB will have access to the final trial dataset.

## Abbreviations

RT: radiation therapy
SRE: skeletal related event
OAR: Organ at risk
IMRT: intensity-modulated radiation therapy
SBRT: stereotactic body radiation therapy
BPI: Brief Pain Inventory
PFS: Pain-free survival
OS: overall survival
NCI: National Cancer Institute
CTCAE: Common Terminology Criteria for Adverse Events
CRF: case report form
SAE: Serious Adverse Event
DSM: Data and Safety Monitoring
DSMC: Data and Safety Monitoring Committee
MID: Minimally Important Difference
RSA: research study assistant

## References

[1] Gomez DR (2016). Local consolidative therapy versus maintenance therapy or observation for patients with oligometastatic non-small-cell lung cancer without progression after first-line systemic therapy: a multicentre, randomised, controlled, phase 2 study. Lancet Oncol.

[2] Nguyen QN (2019). Single-fraction stereotactic vs conventional multifraction radiotherapy for pain relief in patients with predominantly nonspine bone metastases: a randomized phase 2 trial. JAMA Oncol.

[3] Mundy GR (2002). Metastasis to bone: causes, consequences and therapeutic opportunities. Nat Rev Cancer.

[4] Cleeland CS, Ryan KM (1994). Pain assessment: global use of the brief pain inventory. Ann Acad Med Singap.

[5] Steenland E (1999). The effect of a single fraction compared to multiple fractions on painful bone metastases: a global analysis of the Dutch bone metastasis study. Radiother Oncol.

[6] Sze WM (2004). Palliation of metastatic bone pain: single fraction versus multifraction radiotherapy - a systematic review of the randomised trials. Cochrane Database Syst Rev.

[7] Zimmermann C (2014). Early palliative care for patients with advanced cancer: a cluster-randomised controlled trial. Lancet.

[8] Temel JS (2010). Early palliative care for patients with metastatic non-small-cell lung cancer. N Engl J Med.

[9] Hartsell WF (2005). Randomized trial of short- versus long-course radiotherapy for palliation of painful bone metastases. J Natl Cancer Inst.

[10] van der Linden YM (2004). Single fraction radiotherapy is efficacious: a further analysis of the Dutch bone metastasis study controlling for the influence of retreatment. Int J Radiat Oncol Biol Phys.

[11] Chow E (2007). Palliative radiotherapy trials for bone metastases: a systematic review. J Clin Oncol.

[12] Rich SE (2018). Update of the systematic review of palliative radiation therapy fractionation for bone metastases. Radiother Oncol.

[13] Yang, J.C., et al., Radiation for bone metastases: Reconsidering the optimal timing. Journal of Clinical Oncology, 2017. no. 15_suppl: p. 10122–10122.

[14] Lipton A (2000). Pamidronate prevents skeletal complications and is effective palliative treatment in women with breast carcinoma and osteolytic bone metastases: long term follow-up of two randomized, placebo-controlled trials. Cancer.

[15] Saad F (2004). Long-term efficacy of zoledronic acid for the prevention of skeletal complications in patients with metastatic hormone-refractory prostate cancer. J Natl Cancer Inst.

[16] Rosen LS (2004). Long-term efficacy and safety of zoledronic acid in the treatment of skeletal metastases in patients with nonsmall cell lung carcinoma and other solid tumors: a randomized, phase III, double-blind, placebo-controlled trial. Cancer.

[17] Shulman RM (2019). External beam radiation therapy (EBRT) for asymptomatic bone metastases in patients with solid tumors reduces the risk of skeletal-related events (SREs). Ann Palliat Med.

[18] Mirels H (1989). Metastatic disease in long bones. A proposed scoring system for diagnosing impending pathologic fractures. Clin Orthop Relat Res.

[19] Faruqi S (2018). Vertebral compression fracture after spine stereotactic body radiation therapy: a review of the pathophysiology and risk factors. Neurosurgery.

[20] Duran I, Fink MG, Bahl A, Hoefeler H, Mahmood A, Lüftner D, Ghazal H, Wei R, Chung KC, Hechmati G, Green J, Atchison C. Health resource utilisation associated with skeletal-related events in patients with bone metastases secondary to solid tumours: regional comparisons in an observational study. Eur J Cancer Care (Engl). 2017;26(6). 10.1111/ecc.12452. Epub 2016 Feb 10. PMID: 26865392. 10.1111/ecc.12452 26865392

[21] Luftner D (2014). Health resource utilization associated with skeletal-related events in patients with advanced breast cancer: results from a prospective, multinational observational study. Springerplus.

[22] Stopeck A (2012). Cost-effectiveness of denosumab vs zoledronic acid for prevention of skeletal-related events in patients with solid tumors and bone metastases in the United States. J Med Econ.

[23] Langer C, Hirsh V (2010). Skeletal morbidity in lung cancer patients with bone metastases: demonstrating the need for early diagnosis and treatment with bisphosphonates. Lung Cancer.

[24] Cox BW (2012). International spine radiosurgery consortium consensus guidelines for target volume definition in spinal stereotactic radiosurgery. Int J Radiat Oncol Biol Phys.

[25] Bilsky MH (2010). Reliability analysis of the epidural spinal cord compression scale. J Neurosurg Spine.

[26] Khan I (2016). Comparing the mapping between EQ-5D-5L, EQ-5D-3L and the EORTC-QLQ-C30 in non-small cell lung cancer patients. Health Qual Life Outcomes.

[27] Pickard AS (2007). Psychometric comparison of the standard EQ-5D to a 5 level version in cancer patients. Med Care.

[28] Boehling NS (2012). Vertebral compression fracture risk after stereotactic body radiotherapy for spinal metastases. J Neurosurg Spine.

[29] Himelstein AL (2017). Effect of longer-interval vs standard dosing of Zoledronic acid on skeletal events in patients with bone metastases: a randomized clinical trial. JAMA.

[30] Palma DA (2019). Stereotactic ablative radiotherapy for the comprehensive treatment of 4-10 oligometastatic tumors (SABR-COMET-10): study protocol for a randomized phase III trial. BMC Cancer.

[31] Gomez DR (2019). Local consolidative therapy Vs. maintenance therapy or observation for patients with Oligometastatic non-small-cell lung Cancer: long-term results of a multi-institutional, phase II, randomized study. J Clin Oncol.

