# Supplementary material for: Early palliative radiation versus observation for high-risk asymptomatic or minimally symptomatic bone metastases: study protocol for a randomized controlled trial
Source: BMC Cancer. 2020 Nov 17;20:1115. doi: 10.1186/s12885-020-07591-w (PMC7670812; doi:10.1186/s12885-020-07591-w)
Supplement: Supplementary file 1 — Additional file 1. Lesion Identification Worksheet. [file 12885_2020_7591_MOESM1_ESM.docx]

# Patient Name and MRN: Lesions Identification Worksheet

This worksheet is to document and to follow the ≤5 highest risk bone metastases identified at the time of randomization to receive either standard of care (Arm 1) or radiation (Arm 2).

# Highest risk bone metastases definition per protocol:

1. Bulkiest sites of osseous disease ≥ 2cm
2. Disease involving the hip (acetabulum, femoral head, femoral neck), shoulder (acromion, glenoid, humeral head), or sacroiliac joints
3. Disease in long bones with1/3-2/3 cortical thickness (humerus, radius, ulna, clavicle, femur, tibia, fibula, metacarpus, phalanges)
4. Disease in junctional spine (C7-T1, T12-L1, L5-S1) and/or disease with posterior element involvement.

***Example:***

*Lesion location (detailed): left superior acetabulum*

*Highest risk bone metastases definition (1, 2, 3*

*or 4): 2*

*Date of imaging study (within 4 weeks of study entry): 6/11/2017*

*Image study type (CT, PET/CT, MRI): CT*

*Best image series/number (s) to identify the lesion:*

*Screenshot of the imaging study that identifies the lesion and its location (optional):*


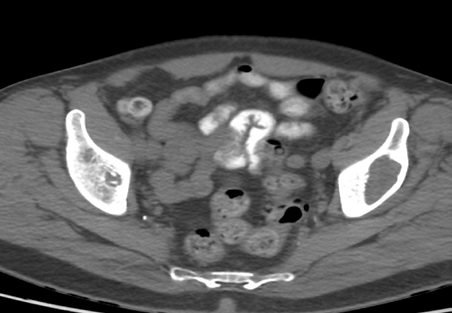


# Lesion 1:

Lesion location (detailed):

Highest risk bone metastases definition (1, 2, 3

or 4):

Date of imaging study (within 4 weeks of study entry):

Image study type (CT, PET/CT, MRI):

Best image series/number (s) to identify the lesion:

Screenshot of the imaging study that identifies the lesion and its location (option

# Lesion 2:

Lesion location (detailed):

Highest risk bone metastases definition (1, 2, 3

or 4):

Date of imaging study (within 4 weeks of study entry):

Image study type (CT, PET/CT, MRI):

Best image series/number (s) to identify the lesion:

Screenshot of the imaging study that identifies the lesion and its location (optional):

# Lesion 3:

Lesion location (detailed):

Highest risk bone metastases definition (1, 2, 3

or 4):

Date of imaging study (within 4 weeks of study entry):

Image study type (CT, PET/CT, MRI):

Best image series/number (s) to identify the lesion:

Screenshot of the imaging study that identifies the lesion and its location (optional):

# Lesion 4:

Lesion location (detailed):

Highest risk bone metastases definition (1, 2, 3

or 4):

Date of imaging study (within 4 weeks of study entry):

Image study type (CT, PET/CT, MRI):

Best image series/number (s) to identify the lesion:

Screenshot of the imaging study that identifies the lesion and its location (optional):

# Lesion 5:

Lesion location (detailed):

Highest risk bone metastases definition (1, 2, 3

or 4):

Date of imaging study (within 4 weeks of study entry):

Image study type (CT, PET/CT, MRI):

Best image series/number (s) to identify the lesion:

Screenshot of the imaging study that identifies the lesion and its location (optional):
