# Supplementary material for: Early palliative radiation versus observation for high-risk asymptomatic or minimally symptomatic bone metastases: study protocol for a randomized controlled trial
Source: BMC Cancer. 2020 Nov 17;20:1115. doi: 10.1186/s12885-020-07591-w (PMC7670812; doi:10.1186/s12885-020-07591-w)
Supplement: Supplementary file 2 — Additional file 2. SRE Assessment Form. [file 12885_2020_7591_MOESM2_ESM.docx]

# IRB 18-196: A Randomized Trial of Early, Upfront Palliative Radiation Therapy versus Standard of Care for Patients with Highest Risk Asymptomatic or Minimally Symptomatic Bone Metastases

**SRE Assessment Form**

| **Patient Name:** | **MRN:** |
| --- | --- |
| **Study ID (if applicable):** | |
| **Date Completed:** | |

1. How many lesions does this participant have enrolled?
2. Is the participant currently taking any opioid medication for pain relating to lesion(s) enrolled?
3. How many SREs is the participant experiencing?
4. Please check off the SREs the participant is currently experiencing:
   - Pathological Fracture
   - Spinal cord compression
   - Palliative Radiation
   - Surgery

Please respond to the questions that pertain to the SREs checked off above.

## Pathological Fracture

- 1. Date of Event: / /
  2. Was the participant hospitalized because of this SRE?
     - Yes
     - No
  3. Date of Hospitalization: / /
  4. Was this SRE identified through imaging?
     - Yes
     - No
  5. Date of Scan (if applicable): _/ /
  6. Is this SRE related to one or more of the lesions enrolled?
     - Yes
     - No
  7. Please identify the related lesion(s): 1. 2. 3. 4. 5.

## Spinal cord compression

- 1. Date of Event: / /
  2. Was the participant hospitalized because of this SRE?
     - Yes
     - No
  3. Date of Hospitalization: / /
  4. Was this SRE identified through imaging?
     - Yes
     - No
  5. Date of Scan (if applicable): _/ /
  6. Is this SRE related to one or more of the lesions enrolled?
     - Yes
     - No
  7. Please identify the related lesion(s): 1. 2. 3. 4. 5.

## Palliative Radiation

- 1. Start Date: / _/
  2. End Date: _/ /
  3. Is this treatment in response to a pathological fracture and/or spinal cord compression?
     - Yes
     - No
  4. Is this SRE related to one or more of the lesions enrolled?
     - Yes
     - No
  5. Please identify the related lesion(s): 1. 2. 3. 4. 5.

## Surgery

- 1. Date of Event: / /
  2. Primary reason for surgery:
     - Severe bone pain
     - Preventative treatment for pathological fracture
     - To treat a pathological fracture
     - Other
  3. If other, please specify:
  4. Is this SRE related to one or more of the lesions enrolled?
     - Yes
     - No
  5. Please identify the related lesion(s):

1.

2.

3.

4.

5.
