# Supplementary material for: Early palliative radiation versus observation for high-risk asymptomatic or minimally symptomatic bone metastases: study protocol for a randomized controlled trial
Source: BMC Cancer. 2020 Nov 17;20:1115. doi: 10.1186/s12885-020-07591-w (PMC7670812; doi:10.1186/s12885-020-07591-w)
Supplement: Supplementary file 3 — Additional file 3. Brief Pain Inventory (BPI) Short Form questionnaire (hyperlink and licensing information). [file 12885_2020_7591_MOESM3_ESM.docx]

Additional file 3. Brief Pain Inventory (BPI) Short Form questionnaire.

Note this form is copyrighted and usage rights can be obtained at: <https://www.mdanderson.org/research/departments-labs-institutes/departments-divisions/symptom-research/symptom-assessment-tools/brief-pain-inventory.html>

MSKCC has purchased an institutional license for the BPI Short Form.
