# Supplementary material for: Early palliative radiation versus observation for high-risk asymptomatic or minimally symptomatic bone metastases: study protocol for a randomized controlled trial
Source: BMC Cancer. 2020 Nov 17;20:1115. doi: 10.1186/s12885-020-07591-w (PMC7670812; doi:10.1186/s12885-020-07591-w)
Supplement: Supplementary file 5 — Additional file 5. EuroQol EQ-5D-5L questionnaire (hyperlink and licensing information). [file 12885_2020_7591_MOESM5_ESM.docx]

Additional file 5. EuroQol EQ-5D-5L questionnaire.

Note this form is copyrighted and usage rights can be obtained at:

<https://euroqol.org/eq-5d-instruments/eq-5d-5l-about/>

MSKCC has officially licensed the EuroQol EQ-5D-5L questionnaire.
