# Supplementary material for: Early palliative radiation versus observation for high-risk asymptomatic or minimally symptomatic bone metastases: study protocol for a randomized controlled trial
Source: BMC Cancer. 2020 Nov 17;20:1115. doi: 10.1186/s12885-020-07591-w (PMC7670812; doi:10.1186/s12885-020-07591-w)
Supplement: Supplementary file 6 — Additional file 6. Adverse Event definitions, process of attribution to radiation therapy, and follow-up. [file 12885_2020_7591_MOESM6_ESM.docx]

**11.1 Adverse Event Reporting**

This study will use the Common Terminology Criteria for Adverse Events (CTCAE) version v4.03 for adverse event (AE) reporting. After informed consent is signed, study site personnel will record the occurrence and nature of each patient’s pre-existing conditions, including clinically significant signs and symptoms of the disease. During the study, site personnel will record any change in the pre-existing condition(s), and the occurrence and nature of any new adverse events. All AEs related to protocol procedures are reported. All AEs occurring after the patient receives the first dose of radiation therapy must be reported in regard to their assessment of the potential relatedness of each AE to protocol procedure, studied disease state, and/or radiation modality via CRF. If a patient’s radiation treatment is discontinued as a result of an AE, personnel must clearly report the circumstances and data leading to any dosage reduction or discontinuation of treatment. Events leading to the clinical outcome of death due to disease progression will be included as part of the safety and efficacy analyses for this study. If a death is considered related to treatment, the death should be reported as a Serious Adverse Event and appropriate guidelines followed for SAE reporting. Any clinically significant findings from labs, vital sign measurements, and other procedures should be reported as well.

**11.2 Definitions of Adverse Events**

**11.2.1 Adverse Event (AE)** Defined as any harm or untoward medical occurrence in a research participant administered a medical product, medical treatment or, procedure even if it does not necessarily have a causal relationship with the product, treatment, or procedure. An adverse event can be any unfavorable and unintended sign (including an abnormal laboratory finding, for example), symptom, or disease temporally associated with the use of a medical product, medical treatment, or procedure, whether or not considered to be related. Resources containing information on AEs include monthly transcripts, assessment forms obtained after each clinic visit, and hospital progress and discharge notes. Grade ≥3 adverse events other than hematologic toxicities will be recorded, graded, and reported appropriately.

**11.2.2 Related or Possibly Related AE**

An AE is “related or possibly related to the research procedures,” if in the opinion of the principal investigator, it is more likely than not caused by the research procedures. AEs that are solely caused by an underlying disease, disorder, or condition of the subject, or by other circumstances unrelated to the research are not “related or possibly related.” If there is any question whether or not an AE is related or possibly related, Grade 3 or higher AE should be reported to the PI and IRB.

**11.2.3 Unexpected AE** An AE is “unexpected” when its nature (specificity), severity, or frequency are not consistent with (a) the foreseeable risk of adverse events associated with the research procedures described in protocol-related documents, such as the IRB approved research protocol, informed consent document, product labeling and package inserts, and; (b) the characteristics of the subject population being studied, including the expected natural progression of any underlying disease, disorder or condition or any predisposing risk factor profile for the adverse event. AEs that do not meet the requirement for expedited reporting will be reported to the IRB as part of the annual renewal of the protocol.

**11.2.4 Attribution to Radiation Therapy** For reporting purposes, attribution is the assessment of the likelihood that an adverse event is caused by the research agent, or protocol intervention. The attribution is assigned by the principal investigator after considering the clinical information, the medical history of the subject, and the past experience with the research agent/intervention.

This is recorded using one of the following five categories:

- Unrelated
- Unlikely Related
- Probably Related
- Possibility Related
- Definitely Related

Related events are those which are most certainly caused by the procedures involved in the research.

Possibly related events are those which may have been caused by the procedures involved in the research.

Not related events are those which are due to an underlying disease, disorder, or condition of the subject, or due to other circumstances unrelated to the research or any underlying disease, disorder, or condition of the subject.

Unknown events are those which have an unclear relationship to the procedures involved in the research, both because more information is needed, and will be provided in follow-up, or because there is no way to make a determination.

**11.3 Follow-up of Adverse Events**

All adverse events will be followed up according to Good Clinical Practice.

During Treatment: Throughout the duration of the study, site personnel will track any change in the condition(s), the occurrence, and the nature of any AEs, and record the highest grade of the adverse event per cycle on the CRF. CTCAE grading will be assigned before each visit for any adverse events experienced during the previous visit period.
