# Supplementary material for: Early palliative radiation versus observation for high-risk asymptomatic or minimally symptomatic bone metastases: study protocol for a randomized controlled trial
Source: BMC Cancer. 2020 Nov 17;20:1115. doi: 10.1186/s12885-020-07591-w (PMC7670812; doi:10.1186/s12885-020-07591-w)
Supplement: Supplementary file 4 — Additional file 4. Cover letter for patient QoL packet. [file 12885_2020_7591_MOESM4_ESM.docx]

# 18-196

**Patient QOL Packet**

Thank you for taking the time to complete this packet. Please complete them to the best of your ability and try to answer all questions.

Surveys in this Packet:

- BPI Short Form
- EQ-5D-5L

**Reminders:**

Please complete the surveys based only on your symptoms in/at your	.

Please be sure to answer #1 and #2 of the BPI Short Form – these questions are frequently missed.
